# Characterization of *Arthrospira platensis NH* Draft Genome

**DOI:** 10.3390/cimb48050461

**Published:** 2026-04-29

**Authors:** Trang Thi Huyen Nguyen, Anh Minh Pham, Linh Khanh Chu, Thuy Thi Kim Dang, Giap Dang Do, Tuan Trong Tran, Chi Nguyen Quynh Ho, Cuong Phan Minh Le, Loan Thi Tung Dang, Nhan Lu Chinh Phan, Son Nghia Hoang, Han Thai Minh Nguyen, Long Thanh Le

**Affiliations:** 1 Institute of Life Sciences, Vietnam Academy of Science and Technology, Ho Chi Minh City 70000, Vietnam; nthtrang@ils.vast.vn (T.T.H.N.); phamminhanh889@gmail.com (A.M.P.); dtkthuy@ils.vast.vn (T.T.K.D.); ddgiap@ils.vast.vn (G.D.D.); tttuan@ils.vast.vn (T.T.T.); hnqchi@ils.vast.vn (C.N.Q.H.); lpmcuong@ils.vast.vn (C.P.M.L.); plcnhan@ils.vast.vn (N.L.C.P.); hnson@ils.vast.vn (S.N.H.); 2 Biotechnology Department, Graduate University of Science and Technology, Vietnam Academy of Science and Technology, Ha Noi 10000, Vietnam; 3 Faculty of Biology and Biotechnology, University of Science, Vietnam National University, Ho Chi Minh 70000, Vietnam; chukhanhlinh97@gmail.com (L.K.C.); dttloan@hcmus.edu.vn (L.T.T.D.); 4 Life Science Department, University of New Hampshire at Manchester, Manchester, NH 03101, USA; han.nguyen@unh.edu

**Keywords:** draft genome, *Arthrospira platensis*, phylogeny

## Abstract

This study aimed to characterize the draft genome of *Arthrospira platensis* NH isolated in Vietnam and evaluate its phylogenetic position within cyanobacteria. Phylogenetic analysis based on 16S rRNA gene sequences confirmed that *A. platensis* NH belongs to the *Arthrospira* clade. The assembled genome comprises 5,548,511 bp and contains 4728 genes, including 4683 protein-coding sequences, 42 tRNA genes, and 3 rRNA genes. Genome mapping revealed conserved gene organization with an overall GC content of 44.45%. A comparative genomic analysis with other *Arthrospira* strains (*A. platensis* NIES-39 and C1) demonstrated high sequence conservation, supporting their close genetic relationships. Secondary structure prediction showed that all 42 tRNA types adopt typical cloverleaf structures, while the ribosomal RNA genes (16S, 23S, and 5S rRNA) exhibit conserved base composition. An analysis of 16S rRNA sequences from 13 cyanobacterial taxa identified 230 polymorphic sites, providing informative markers for phylogenetic divergence among genera. Collectively, these results establish a comprehensive genomic and phylogenetic framework for *A. platensis* NH and provide insights into its genetic relationships and potential biotechnological applications.

## 1. Introduction

The cyanobacterium *Arthrospira platensis* is widely recognized for its exceptionally high contents of proteins, essential amino acids, vitamins, and photosynthetic pigments, making it a valuable nutritional resource for both humans and animals [1]. Naturally occurring in soda lakes, *Arthrospira* is commonly cultivated in large-scale open ponds under strongly alkaline conditions, which effectively limit microbial contamination and favor stable biomass production [2]. Unlike many plant-based food sources, *Arthrospira*-derived products retain much of their nutritional value even after exposure to high-temperature processing [3,4,5]. These characteristics have stimulated growing interest in the genomic features of *A. platensis*, particularly in relation to its biotechnological and environmental applications.

Although the cyanobacterium *Arthrospira platensis* (widely known under the commercial name “Spirulina”) is one of the most extensively studied cyanobacteria due to its economic importance, its taxonomy has historically been controversial. In an important previous taxonomic revision of the genus *Arthrospira*, Komárek and Lund (1990) reclassified planktonic forms of *Spirulina* (such as *S. maxima* and *S. fusiformis*) into the genus *Arthrospira* [6]. More recently, studies have partially resolved these taxonomic problems by separating commercially grown taxa into a newly established genus. Specifically, Nowicka-Krawczyk et al. (2019) demonstrated that *Arthrospira* and the newly erected genus *Limnospira* are distinct monophyletic genera [7]. These two genera can be morphologically distinguished from each other by the presence of aerotopes in *Limnospira*, providing a clear basis for transferring aerotope-bearing *Arthrospira* strains into this new genus [7]. Recent studies have further refined this taxonomy by integrating molecular, morphological, and ecological data, supporting the reassignment of *Arthrospira platensis* to *Limnospira platensis* and highlighting the ecological specialization of these taxa in alkaline and saline environments [8].

The genome of *Arthrospira platensis* has been widely characterized, exhibiting sizes ranging from approximately 5.5 Mb in local isolates to nearly 6.8 Mb in reference strains such as NIES-39, with a relatively conserved GC content of around 44% [9,10]. These genomes typically consist of a single circular chromosome harboring 4500–6500 predicted protein-coding genes, 40–50 tRNA genes, and multiple rRNA operons. While the core genomic architecture is highly conserved, strain-specific genomic regions and islands contribute to variability in metabolic pathways, pigment biosynthesis, and tolerance to environmental stressors [11,12].

The previous study showed that the complete genome decoding of the reference strain NIES-39 has identified a genome size of approximately 6.8 Mb, characterized by an abundance of repetitive elements and group II introns [3]. Furthermore, comparative genomic analyses across various strains—such as the YZ strain or the O9.13F strain isolated from alkaline, winter-freezing lakes in Siberia—have highlighted remarkable genome plasticity and genetic diversity, primarily driven by lateral gene transfer and sequence duplication [13]. Most recently, in-depth studies have focused on mapping the methylome and identifying core genetic defense systems of *L. platensis*, including restriction-modification (R-M) and CRISPR-Cas systems, aiming to overcome barriers to genetic engineering in this organism [14]. Despite the wealth of global genomic data, publications regarding the genome sequences of indigenous strains isolated from tropical waters in Vietnam remain limited, thereby affirming the necessity and urgency of this research [8].

In Vietnam, *Arthrospira* has been extensively cultivated for food and nutritional purposes; however, to date, no complete or draft genome sequence originating from Vietnamese *A. platensis* strains has been publicly deposited. In this study, we report the draft genome sequence of *Arthrospira platensis* NH, isolated from central Vietnam. Furthermore, we assessed its phylogenetic placement using 16S rRNA analysis and provided comparative genomic insights into features underlying its physiology, environmental adaptation, and biotechnological potential.

## 2. Materials and Methods

### 2.1. Culture Conditions and Maintenance

The sample of *Arthrospira platensis* NH was collected from Ninh Hoa city, Khanh Hoa Province, Vietnam. Morphological characteristics and cell viability were evaluated using a Cytell fluorescence microscope (GE Healthcare, Arlington Heights, IL, USA), confirming the spiral structure and high viability of this *Arthrospira platensis* strain.

*Arthrospira platensis NH* cultures were maintained in alkaline nutrient medium based on Zarrouk’s formulation, adjusted to maintain high pH and support robust growth under controlled laboratory conditions [15]. Cultures were incubated at an optimal temperature of 30 °C under a 2000 lux light regime, with a light:dark period of 12:12 h, with gentle mixing on an orbital shaker at 100 RPM to maintain a homogeneous suspension and prevent settling. To maintain physiological stability, cultures were subcultured every 5–7 days upon reaching an OD_680nm_ of 1.0, using a 10% (*v*/*v*) inoculum in fresh medium. Culture integrity and growth performance were periodically monitored [16].

### 2.2. DNA Extraction and Sequencing

Genomic DNA of *Arthrospira platensis* NH was extracted using the phenol–chloroform method following standard protocols. DNA quality and purity were assessed using a NanoDrop spectrophotometer (Thermo Fisher Scientific, Inc., Waltham, MA, USA), yielding an A260/280 ratio of 1.82. DNA concentration was quantified using a Qubit fluorometer (Thermo Fisher Scientific, Inc., Waltham, MA, USA) and measured at 66 ng/µL. Fragment size distribution was evaluated using a TapeStation system (Agilent Technologies, Santa Clara, CA, USA), which indicated high-molecular-weight DNA with an average fragment length of approximately 20 kb, suitable for paired-end library preparation. Sequencing libraries were prepared using the Illumina Nextera XT DNA Library Preparation Kit (Illumina, Inc., San Diego, CA, USA) according to the manufacturer’s instructions. Paired-end sequencing (2 × 150 bp) was performed on the Illumina NovaSeq 6000 platform. The sequencing run generated high-quality reads corresponding to an estimated average genome coverage of approximately 150×.

### 2.3. De Novo Assembly

Genomic DNA was sequenced on the Illumina platform, and raw sequencing data were initially processed with fastp v0.23.1 [17] to remove adapters, trim low-quality bases, and filter reads containing uncertain or unidentified nucleotides (“N”), both pre- and post-cleanup read summaries indicated a %Q30 > 80%, confirming the data is of sufficient quality to initiate de novo assembly. The resulting clean reads were then assembled de novo using Unicycler v0.4.8 [18]. Assembly quality was evaluated with QUAST v5.2.0 [19]. The assembly was further validated, and sequencing reads were aligned back to the contigs to identify unusually low-coverage regions that could indicate potential misassemblies. Genome completeness and contamination were assessed using CheckM v1.2.1 [20]. ANI calculations were performed using FastANI v1.34 with default fragment length (1 kb) and minimum alignment thresholds, following the standard FastANI workflow [21]. BUSCO v5.5.0 was run in genome mode with default parameters on the draft assembly [22].

### 2.4. Bioinformatics Analysis

The assembled genome of *Arthrospira platensis* NH was annotated using Prokka v1.14.6 [23], a pipeline optimized for the rapid and accurate annotation of prokaryotic genomes. Parameters were adjusted according to the genomic features of cyanobacteria to ensure accurate gene prediction and functional assignment. GTDB-Tk (v2.1.1) [24] was used exclusively for genome-based taxonomic assignment of strain NH within the Genome Taxonomy Database framework. This analysis was performed to confirm species-level classification and was not intended for genome-wide phylogenomic tree reconstruction.

The graphical genetic map of *Arthrospira platensis* NH was generated using CGView [25]. By integrating the assembled genome sequence with the annotation file obtained from Prokka, gene positions and functional categories were extracted and visualized in a circular genome atlas. This atlas highlights essential genomic features, including coding sequences, RNA genes, nucleotide composition, GC content, and GC skew, thereby providing a comprehensive overview of the genome organization and intrinsic structural properties of the DNA. In addition, the secondary structures of transfer RNAs (tRNAs) were predicted using tRNAscan-SE v2.0 [26]. This tool allowed for the accurate detection of tRNA genes and characterization of their canonical cloverleaf secondary structures.

All predicted genes in the *Arthrospira platensis* NH genome were functionally annotated using eggNOG-mapper [27], which leverages the eggNOG database of orthologous groups for large-scale functional inference. This approach enabled comprehensive annotation by assigning genes to Clusters of Orthologous Groups (COGs) and predicting Gene Ontology (GO) terms, KEGG pathways, and protein domains (e.g., Pfam) [28].

### 2.5. Phylogenetic Analysis

To determine the phylogenetic position of *Arthrospira platensis* NH and evaluate its genetic relationships relationship with other *Arthrospira* species, the 16S rRNA gene sequence was aligned with reference sequences retrieved from the GenBank database. Multiple sequence alignment was performed using the ClustalW algorithm implemented in MEGA v12 [29,30]. Phylogenetic reconstruction was carried out using the maximum-likelihood method with the Tamura–Nei substitution model [31,32]. Tree topology robustness was assessed with 1000 bootstrap replicates [33]. Moreover, the phylogenetic tree was reconstructed using the Bayesian inference method implemented in the MrBayes program (v3.2.6) [34]. The analysis was performed under the GTR nucleotide substitution model, as selected by model testing. The Markov Chain Monte Carlo (MCMC) analyses were performed with four chains (one cold and three heated) [35]. The Markov Chain Monte Carlo analyses were run for 10,000,000 generations, with trees sampled every 1000 generations. The initial 25% of sampled trees were discarded as burn-in. In addition, sequence variation sites within the *A. platensis* NH 16S rRNA gene were identified by a comparison with homologous sequences from other members of the genus *Arthrospira*.

## 3. Results

### 3.1. Classification and Features

**Figure 1:**
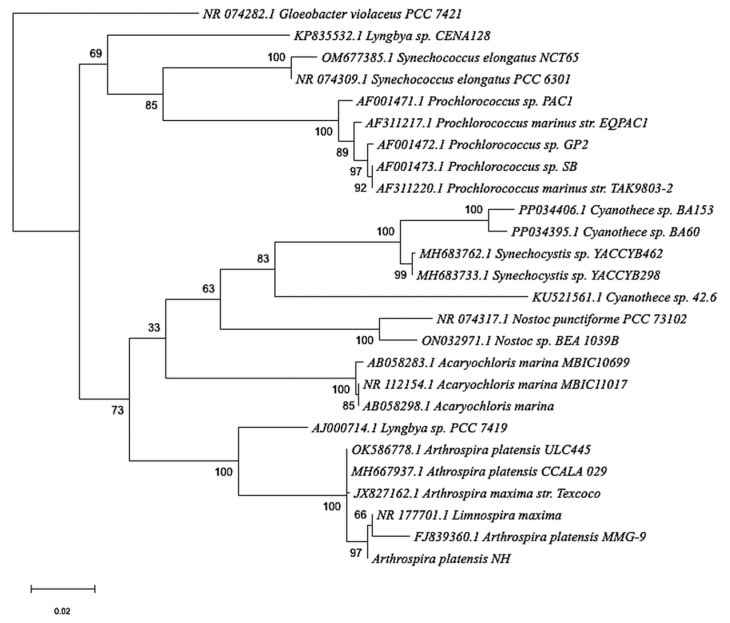
Maximum likelihood tree based on 16S rRNA gene sequences showing the clustering of *Arthrospira platensis* NH among representative cyanobacterial taxa. Bootstrap support values (%) based on 1000 replicates are shown at the nodes. The scale bar represents 0.02 substitutions per nucleotide position. *Gloeobacter violaceus* PCC 7421 was used as the outgroup.

**Figure 2:**
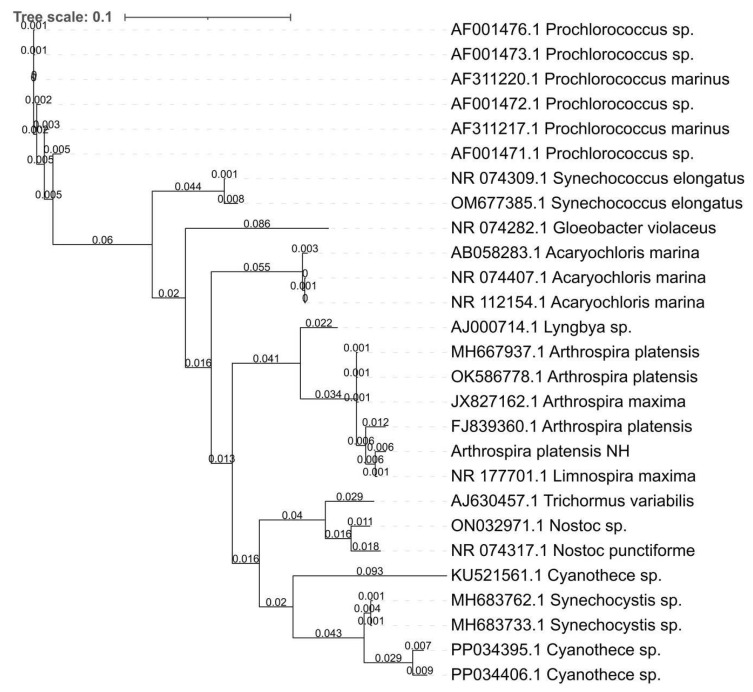
Bayesian inference phylogenetic tree constructed from 16S rRNA gene sequences, illustrating the phylogenetic position of *Arthrospira platensis* NH within representative cyanobacterial taxa. The scale bar corresponds to 0.1 substitutions per nucleotide position.

*Arthrospira* species are filamentous cyanobacteria characterized by multicellular, cylindrical trichomes forming open, left-handed helical structures (Table 1). The Maximum likelihood and Bayesian inference phylogenetic trees based on 16S rRNA gene sequences show that *Arthrospira platensis* NH clusters with reference *Arthrospira* strains (Figure 1 and Figure 2). The trees illustrate sequence similarity and clustering patterns among selected cyanobacterial taxa.

**Figure 3:**
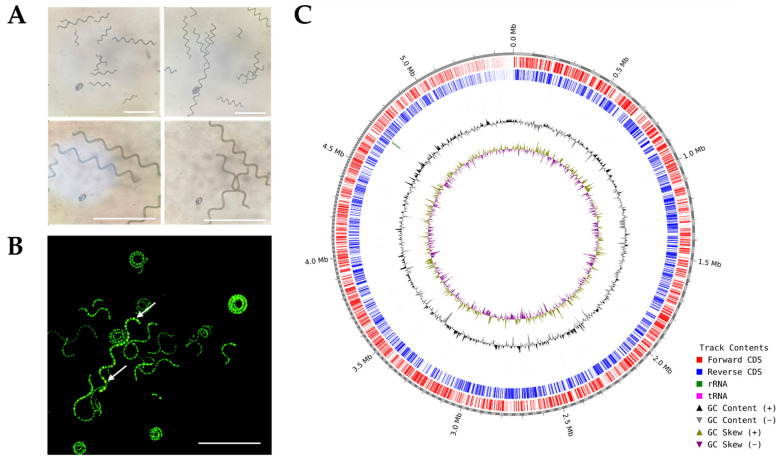
Microphotographs of *Arthrospira platensis* NH and a visual representation of its draft genome. (**A**): Morphology of *Arthrospira platensis* NH. (**B**): Live/dead staining indicates live cells (green) and dead cells (yellow; white arrow); scale bar: 50 µm. (**C**): Graphical representation of contig-based draft genome. From outside to center: genes on forward strand, genes on reverse strand, RNA genes (rRNAs green; tRNAs pink), GC skew, GC content.

A microscopic examination confirmed that *A. platensis* NH exhibits a characteristic spiral morphology. Live/dead staining further demonstrated a high proportion of viable cells in the culture (Figure 3A,B), indicating that the strain used for genome sequencing was in good physiological condition.

### 3.2. Genome Sequencing and Annotation

#### 3.2.1. Genome Sequencing and Assembly

The de novo assembly of the *A. platensis* NH genome resulted in a total assembled length of 5,548,511 bp contributed to 1352 contigs (Table 2) and the distribution of genes across general COG functional categories (Table 3). The largest contig measured 90,760 bp, accounting for approximately 1.64% of the total assembly length. Assembly statistics yielded an N50 value of 20,730 bp and an L50 value of 82, reflecting a moderately fragmented draft genome. A total of 4728 genes were predicted, including 4683 protein-coding sequences (CDSs), 42 tRNA genes, and 3 rRNA genes. A genome quality assessment using CheckM indicated high completeness (98.9%) with low contamination (0.6%). BUSCO analysis based on the cyanobacteria_odb10 lineage further supported the high quality of the assembly, with 98.9% complete BUSCOs, including 97.7% single-copy and 1.2% duplicated genes.

#### 3.2.2. Genome Properties

A graphical genome atlas of *A. platensis* NH was constructed using CGView to visualize genome organization (Figure 3C). The circular map illustrated the distribution of coding sequences on the forward and reverse strands, the locations of RNA genes (rRNAs in green and tRNAs in pink), GC content, and GC skew. Despite the fragmented nature of the assembly, the genome exhibited a conserved organization pattern consistent with other *Arthrospira* strains. The GC content remained relatively stable across the genome, with an average value of approximately 44.45%, suggesting strong compositional conservation. GC skew analysis revealed balanced nucleotide distribution between replichores, consistent with patterns reported in other cyanobacterial genomes.

A comparative genomic analysis was performed between *A. platensis* NH and two reference strains, *A. platensis* C1 and *A. platensis* NIES-39 (Table 4). The NH genome is smaller in size (5.55 Mb) compared to C1 (6.09 Mb) and NIES-39 (6.79 Mb), yet it retains a high proportion of functionally annotated genes.

An average nucleotide identity (ANI) analysis confirmed a close genetic relationship among these strains. *A. platensis* NH shares 99.28% ANI with strain C1 and 94.19% ANI with strain NIES-39, indicating particularly high genomic similarity with C1. Functional annotation based on COGs, KEGG pathways, and Pfam domains showed that conserved genes are predominantly associated with essential metabolic processes, membrane transport, signal transduction, and stress response mechanisms.

In addition, an antiSMASH analysis identified several biosynthetic gene clusters, including terpene- and RiPP-like clusters. Some of these clusters exhibited divergence relative to those found in strains C1 and NIES-39, suggesting the presence of strain-specific metabolic features that may contribute to environmental adaptation and biotechnological potential.

#### 3.2.3. RNAs Structure

**Figure 4:**
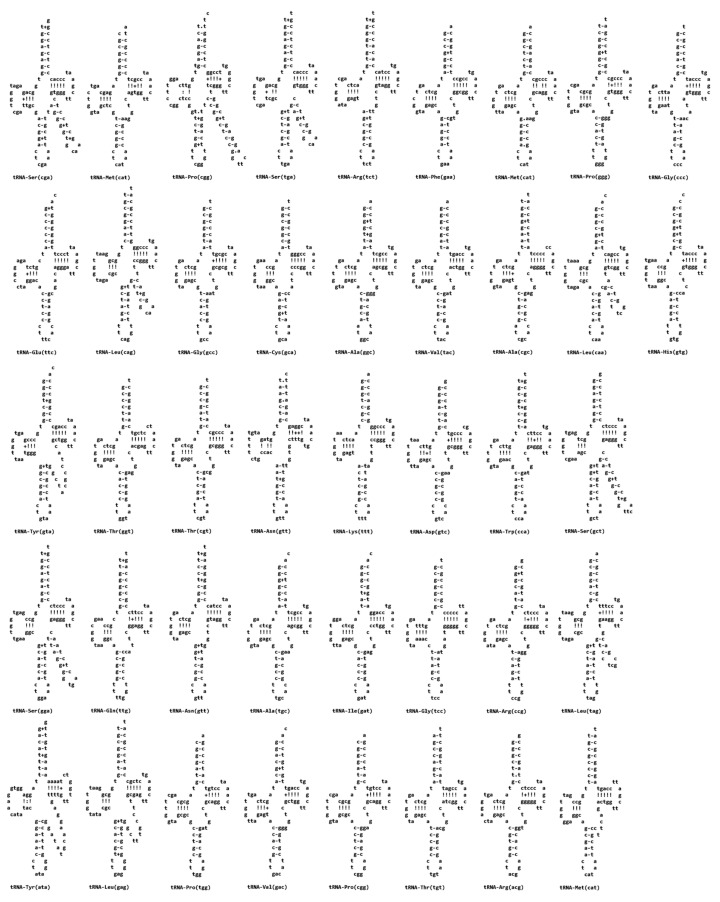
tRNA structures of *Arthrospira platensis* NH. The analysis of 16S RNA sequences of 13 samples (including 5 *Arthrospira*, 1 *Limnospira*, 1 *Lyngbya*, 1 *Nostoc*, 1 *Synechocystis*, 1 *Cyanothece*, 1 *Acaryochloris*, 1 *Synechococcus*, and 1 *Prochlorococcus*) showed 230 polymorphic sites (Figure 5). These variations reflect genetic divergence among genera and species, especially between *Arthrospira* and other more distantly related cyanobacteria. However, sequences within the *Arthrospira* group were highly conserved, supporting their close genetic relationship. The presence of some conserved motifs across multiple genera also highlights shared ancestral traits, while specific substitutions and indels (insertions/deletions) serve as potential markers for phylogenetic discrimination and strain identification. The “+” symbol is used to indicate the specific non-canonical G-T pairing.

A total of 42 tRNA genes were identified in the *A. platensis* NH genome. Secondary structure prediction showed that all tRNA genes can be folded into canonical cloverleaf structures (Figure 4). The length of the tRNA genes ranged from 71 bp (tRNA-Cys) to 98 bp (tRNA-Pro), with GC content varying from 38.4% (tRNA-Tyr) to 64.5% (tRNA-Pro). The genome also contained three ribosomal RNA genes, including 16S rRNA (1485 bp), 23S rRNA (2880 bp), and 5S rRNA (109 bp). The A + T contents of the 16S, 23S, and 5S rRNA genes were 44.3%, 46.2%, and 46.8%, respectively, indicating conserved nucleotide composition across ribosomal components.

**Figure 5:**
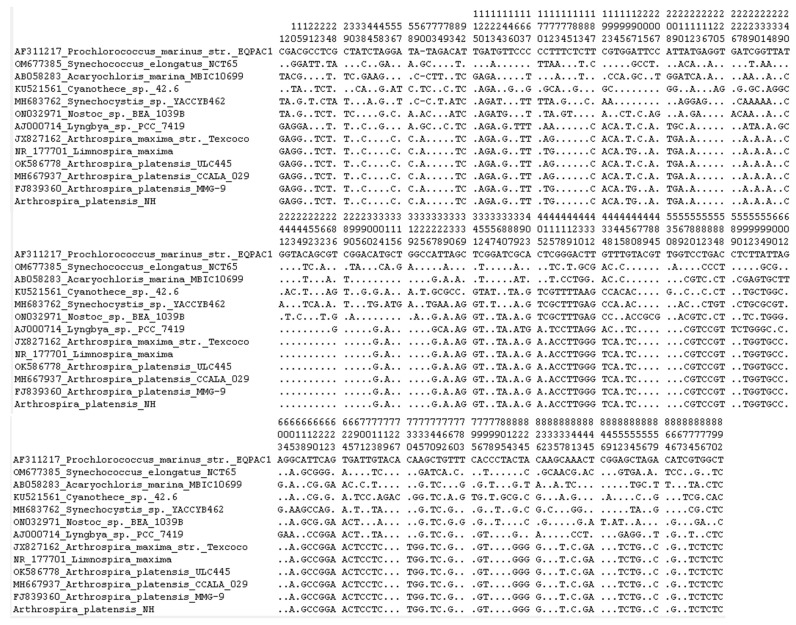
Variable positions of 16S-RNA sequences from *Arthrospira platensis* strains. Sequence identities are indicated by dots.

## 4. Discussion

Studies on Vietnamese *Arthrospira* (now largely reclassified as *Limnospira*) have focused on cultivation, biomass production, and biotechnology. Since its introduction in the 1970s, research has emphasized optimizing growth, reducing costs, and improving yield for food and aquaculture uses [43,44,45]. However, phylogenetic research on Vietnamese strains remains limited. A few studies have applied molecular identification (e.g., 16S rRNA analysis, or Multiplex RAPD-PCR) to confirm species such as *A. platensis* from local habitats, but comprehensive phylogenetic analyses are still scarce [46,47]. Available evidence suggests that Vietnamese strains likely cluster within the globally distributed *Limnospira*/*Arthrospira* clade, which has been redefined using taxonomy and molecular data.

In this study, genomic characterization combined with targeted sequence-based analyses provides an overview of the genomic features of *Arthrospira platensis* NH within the current taxonomic framework of *Arthrospira* and related genera. Taxonomic assignment was conducted using GTDB-Tk, and a maximum likelihood analysis of the 16S rRNA gene was performed to support genus-level classification. The 16S rRNA tree is presented to illustrate sequence similarity and clustering patterns among selected cyanobacterial taxa and is not intended to represent a comprehensive phylogenetic inference.

Morphological observations confirmed the characteristic spiral trichome structure and high cell viability of strain NH, consistent with previous studies indicating that helicoidal morphology contributes to motility, light utilization, and ecological fitness in alkaline aquatic environments. Such structural traits are widely regarded as adaptive features that enhance survival and physiological performance under fluctuating environmental conditions [48]. Phylogenetic reconstruction based on the 16S rRNA gene placed *A. platensis* NH firmly within the *Arthrospira* clade with strong bootstrap support, while also indicating a close genetic relationship with *Limnospira*. This result is consistent with recent taxonomic revisions based on genome-scale comparative frameworks, which recognize *Arthrospira* and *Limnospira* as distinct yet closely related genera. Importantly, the agreement between 16S rRNA-based phylogenetic placement and GTDB-Tk-based taxonomic classification supports the robustness of the species assignment for strain NH.

The assembled genome (≈5.55 Mb, ~4728 genes) shows strong similarities in sequence composition and gene content with *A. platensis* NIES-39 and C1, consistent with comparative genomics that reported high conservation among sequenced *Arthrospira* strains [49]. High ANI values, especially the 99.28% identity with strain C1, indicate a very close genetic relationship, while the smaller genome size of NH suggests potential genome streamlining. Genome size variation and potential genome reduction have been reported among *Arthrospira/Limnospira* strains and are often interpreted as adaptive responses to specific ecological conditions, involving the selective retention of core metabolic functions and the loss of dispensable genomic regions [12,14]. This pattern has been observed in other cyanobacterial lineages inhabiting stable yet extreme environments, such as alkaline or saline waters. The relatively stable GC content (~44–45%) across the NH genome further supports strong compositional conservation and may contribute to maintaining DNA stability under high-pH conditions. Functional annotation revealed that a large proportion of conserved genes are associated with energy metabolism, membrane transport, signal transduction, and stress response pathways. These functional categories are consistent with the metabolic flexibility required for survival in alkaline and nutrient-variable environments and align with previous studies demonstrating the ability of *Arthrospira* strains to adjust their metabolism under salt stress, mixotrophic cultivation, or alternative nutrient sources [14].

Additional genomic features—including a complete set of 42 tRNAs, structurally conserved rRNAs, and well-maintained translational machinery—indicate strong genetic stability and functional robustness. Furthermore, a 16S rRNA polymorphism analysis across 13 cyanobacterial taxa revealed 230 informative variation sites, which support phylogenetic discrimination among genera while highlighting the high sequence conservation characteristic of *Arthrospira* [4].

Notably, biosynthetic gene cluster (BGC) analysis revealed the presence of terpene- and RiPP-like pathways in *Arthrospira platensis* NH, some of which show divergence in gene composition and organization when compared with reference strains such as C1 and NIES-39. Terpene-related genes, including homologs involved in phytoene and carotenoid biosynthesis, have been previously identified in *Arthrospira* and *Limnospira* genomes and are known to play important roles in pigmentation, photoprotection, and oxidative stress mitigation in alkaline environments [50]. Comparative genomic analyses further suggest that variation within these terpene-associated pathways may contribute to strain-specific metabolite profiles and adaptive strategies among geographically distinct *Arthrospira* isolates [51].

In parallel, RiPP-like gene clusters, which are increasingly recognized as a source of bioactive peptides in cyanobacteria, may expand the secondary metabolic repertoire of strain NH, although their functional roles require experimental validation. Beyond secondary metabolism, genes associated with stress adaptation—including heat shock proteins, redox homeostasis systems, and osmotic stress response pathways—have been shown to be transcriptionally regulated under environmental stress conditions in *Arthrospira*, highlighting their importance for cellular resilience [52]. The coexistence of a conserved core metabolic framework with divergence in secondary metabolite and stress-response gene clusters suggests that *A. platensis* NH retains the canonical genomic architecture of the genus while harboring locally shaped traits. These genomic features are likely the result of long-term adaptation to the specific physicochemical conditions of alkaline aquatic systems in central Vietnam and may underlie strain-specific physiological performance and biotechnological potential.

In this study, *A. platensis* NH was characterized using a polyphasic approach integrating morphological, molecular, and genome-based analyses. Morphological observations confirmed the typical spiral trichome structure, while 16S rRNA phylogeny consistently placed the strain within the *Arthrospira* clade. Genome-based analyses provided stronger support, with Genome Taxonomy Database classification and high Average Nucleotide Identity values (99.28% with strain C1) confirming species-level assignment. Comparative genomics revealed conserved genome architecture and functional profiles, alongside variation in biosynthetic gene clusters, suggesting strain-specific adaptive potential. The concordance among these datasets highlights the robustness of the polyphasic framework for cyanobacterial taxonomy.

Overall, the genomic characterization of *A. platensis* NH provides a valuable reference for understanding the genomic features, ecological context, and functional potential of *Arthrospira* strains. The presence of conserved core genes together with strain-specific genomic variations suggests potential adaptive diversity within the genus and supports its continued relevance in biotechnology, nutrition, and environmental applications. Future studies integrating transcriptomic, proteomic, and metabolomic data will help to connect genomic content with functional expression and further clarify mechanisms underlying stress response and metabolic performance.

## 5. Conclusions

*A. platensis* NH presents a highly complete and well-annotated draft genome, representing the first genomic resource for this species originating from Vietnam. Comparative genomic analyses, together with genome-based taxonomic assignment and 16S rRNA-based phylogenetic assessment, indicate strong conservation with globally reported *Arthrospira/Limnospira* lineages while also revealing features associated with environmental tolerance and metabolic diversity. This genomic resource provides a solid foundation for future studies on cyanobacterial genetics, biotechnology, strain improvement, and optimization of industrial cultivation.

## Figures and Tables

**Table 1:** Classification and general features of *A. platensis* NH.

Property	Term	References
Current classification	Domain Bacteria	TAS [36]
	Phylum Cyanobacteria	TAS [37,38]
	Order Oscillatoriales	TAS [37,38]
	Family Microcoleaceae	TAS [39]
	Genus *Arthrospira*	TAS [39,40]
	Species *Arthrospira platensis* NH	TAS [38]
Gram stain	Negative	TAS [41]
Filament morphology	Spiral	TAS [41]
Gliding motility	None	
Sporulation	None	
Temperature range	20–40 °C	TAS [41]
Optimum temperature	30–35 °C	TAS [41]
pH	8.0–10.0	TAS [42]
Salinity	0.06 g/L	TAS [42]
Draft genome size	5.548 Mb	IDA
Biotic relationship	Free living	NAS
Geographic location	Ninh Diem Ward, Ninh Hoa City, Khanh Hoa province, Vietnam	NAS

Evidence codes—IDA: inferred from direct assay (first time in publication); TAS: traceable author statement (i.e., a direct report exists in the literature); NAS: non-traceable author statement (i.e., not directly observed for the living, isolated sample but based on a generally accepted property for the species or anecdotal evidence).

**Table 2:** De novo assembly result.

Attributed	Value
Genome size (bp)	5,548,511
Number of contigs	1352
G + C content (bp)	44.45%
Genes (total)	4728
CDSs (total)	4683
The longest contig (bp)	90,760
tRNAs	42
rRNAs	3
N50 value	20,730
L50 value	82

Note: N50 is defined as the shortest contig length such that the sum of contigs of equal or greater length will cover 50% of the total assembly. L50 is defined as the smallest number of contigs whose total length is half the genome size.

**Table 3:** Number of genes associated with the general COG functional categories.

Code	Value	% Value	Description
A	5	0.114%	RNA processing and modification
B	1	0.023%	Chromatin structure and dynamics
C	197	4.485%	Energy production and conversion
D	65	1.480%	Cell cycle control, cell division, chromosome partitioning
E	176	4.007%	Amino acid transport and metabolism
F	71	1.617%	Nucleotide transport and metabolism
G	112	2.550%	Carbohydrate transport and metabolism
H	152	3.461%	Coenzyme transport and metabolism
I	68	1.548%	Lipid transport and metabolism
J	164	3.734%	Translation, ribosomal structure, and biogenesis
K	119	2.709%	Transcription
L	305	6.944%	Replication, recombination, and repair
M	245	5.578%	Cell wall/membrane/envelope biogenesis
N	10	0.228%	Cell motility
O	175	3.985%	Post-translational modification, protein turnover, chaperones
P	152	3.461%	Inorganic ion transport and metabolism
Q	69	1.571%	Secondary metabolite biosynthesis, transport, and catabolism
S	1057	24.066%	Function unknown
T	295	6.717%	Signal transduction mechanisms
U	42	0.956%	Intracellular trafficking, secretion, and vesicular transport
V	86	1.958%	Defense mechanisms
-	562	12.796%	Not in COG

**Table 4:** Genome statistics comparison among *Arthrospira* spp.

Genome Name	*A. platensis* NH	*A. platensis* C1	*A. platensis* NIES-39
Genome size (bp)	5,548,511	6,089,210	6,788,435
Total genes	4728	6153	6676
Protein-coding genes	4683	6108	663
tRNA genes	42	45	46
Kegg pathways	1110	1012	993
% Kegg pathways	23.48%	16.45%	14.87%
Kegg Orthology (KO)	1925	1837	1702
% Kegg Orthology (KO)	40.71%	29.86%	25.49%
COGs	3830	3459	357
%COGs	81.01%	56.22%	53.48%
Pfam	3633	3529	3598
%Pfam	76.84%	57.35%	53.89%

## Data Availability

The original contributions presented in this study are included in the article. Further inquiries can be directed to the corresponding author.
